# Controlling bacterial biofilm formation by native and methylated lupine 11S globulins

**DOI:** 10.3389/fmicb.2023.1259334

**Published:** 2023-09-26

**Authors:** Gamal Enan, Seham Abdel-Shafi, Mona El-Nemr, Wesam Shehab, Ali Osman, Mahmoud Sitohy, Basel Sitohy

**Affiliations:** ^1^Department of Botany and Microbiology, Faculty of Science, Zagazig University, Zagazig, Egypt; ^2^Department of Chemistry, Faculty of Science, Zagazig University, Zagazig, Egypt; ^3^Department of Biochemistry, Faculty of Agriculture, Zagazig University, Zagazig, Egypt; ^4^Department of Clinical Microbiology, Infection and Immunology, Umeå University, Umeå, Sweden; ^5^Department of Radiation Sciences, Oncology, Umeå University, Umeå, Sweden

**Keywords:** 11S globulin, methylated, antibiofilm activity, pathogenic bacteria, FTIR

## Abstract

The antibacterial and anti-biofilm activities of the 11S globulins isolated from lupin seeds (*Lupinus termis*), and its methylated derivative (M11S), were investigated against seven pathogenic gram-positive and gram-negative bacteria. The MIC of 11S ranged from 0.1 to 4.0 μg/ml against 0.025 to 0.50 μg/ml for M11S, excelling some specific antibiotics. The MICs of M11S were 40–80 times lower than some specific antibiotics against gram-positive bacteria and 2–60 times lower than some specific antibiotics against gram-negative bacteria. One MIC of 11S and M11S highly reduced the liquid growth of all tested bacteria during 24 h at 37°C. They also inhibited biofilm formation by 80%−86% and 85%−94%, respectively (gram-positive), and 29%−44% and 43%−50%, respectively (gram-negative). M11S prevented biofilm formation by gram-positive bacteria at minimum biofilm inhibitory concentration (MBIC), 0.025–0.1 μg/ml against 0.1–0.5 μg/ml for gram-negative bacteria, i.e., 4–20 times and 4–7 times anti-biofilm inhibitory action compared with 11S, respectively. Biofilm formation of two bacteria revealed no adhered cells on glass slides for 24 h at 37°C, i.e., was entirely prevented by one MBIC of 11S and M11S. Scanning electron microscopy indicated microbial biofilm deformation under the action of 11S and M11S, indicating their broad specificity and cell membrane-targeted action.

## 1 . Introduction

Antibiotic resistance has reached world records, dangerously threatening public health (Gonzalez-Zorn, 2021). Biofilm-forming bacteria, e.g., *Pseudomonas aeruginosa* and *Staphylococcus aureus*, are among the most virulent and problematic pathogens, causing life-threatening hazards. Biofilms can protect bacterial species from environmental challenges, e.g., nutrient depletion and the threat of antibiotics (Yarwood et al., 2004), as a consequence of forming dormant permanent cells resisting conventional antibiotics. Therefore, there is a crucial demand for novel treatment strategies affecting resistant strains and persisting bacterial biofilms. Antimicrobial peptides (AMPs) have successfully recorded inhibitory actions against bacterial biofilm before or after its formation (Dawgul et al., 2014).

AMPs appeared as promising antiplaque agents preventing bacterial colonization at early biofilm developmental stages (Hancock and Lehrer, 1998). AMPs are already ancient in origin and ubiquitous. They are naturally produced by bacteria, plants, vertebrates, and invertebrates (Altman et al., 2006) and are essential to human innate immunity. Recently, AMPs have drawn attention as an alternative class of antimicrobials with high selectivity for prokaryotes while exhibiting minimal emergence of microbial resistance (Concannon et al., 2003). In addition, AMPs were reported to reduce biofilm formation at later stages (Gilmore et al., 2009).

For several years, basic proteins, spices, herbs, and herbal extracts with antimicrobial activities have been thoroughly and widely discussed (Abdel-Shafi et al., 2019b; Imbabi et al., 2021; Osman et al., 2021; Ebrahim et al., 2022; Mahmoud et al., 2023). Plant biodiversity provides an essential source of chemical compounds with variable and diversified multiple therapeutic applications, including antiviral, antibacterial, antifungal, and anticancer activities (Osman et al., 2013). Legumes represent an essential component of the traditional diets of many regions worldwide (Ebert, 2014). Principally, lupin seeds are distinctly characterized by a virtually absent starch and high protein content compared with other legumes such as beans and peas (Torres et al., 2005). Globulins and albumins are the two main protein fractions in lupin seeds, present at a 9:1 quantitative ratio. Lupin globulin consists of two major subunits called α-conglutin (11S and “legumin-like”) and β-conglutin (7S and “vicilin-like”), accounting for ~33 and 45% of the total protein content in i, respectively (Pereira et al., 2022).

Several strategies have been suggested to enhance the antimicrobial activities of proteins, including chemical modification to obtain new cationic proteins. Esterification is an effective and easy tool for protein modification that blocks free carboxyl groups, thus elevating the net positive charge and rendering the modified protein that is more basic and biologically active, especially as an antimicrobial agent (Sitohy et al., 2001; Sitohy and Osman, 2010). However, these modified cationic proteins have never been tested for their potential as anti-biofilm agents. It is hypothesized that these positively charged esterified products may have two mechanistic pathways in counteracting bacterial biofilm formation. The first pathway is through their direct attack on bacterial viability, affecting consequently their ability to form biofilms. The second pathway may act by competing for the negatively charged solid surfaces, either glass or metallic, by their positively charged chemical characteristics, thus affecting bacterial adhesion and biofilm formation. Therefore, this study aimed to test this hypothesis and evaluate the esterified lupin seed globulin (M11S) capacity to counteract bacterial biofilm formation compared with its native form (11S).

## 2 . Materials and methods

### 2.1 . Isolation, chemical characterization, and esterification of lupine proteins

#### 2.1.1 . Sample preparation, protein isolation, and 11s globulin from lupin seed isolation

Lupine seeds (*Lupinus termis*), obtained from a local market in Zagazig City, Sharkia, Egypt, were thoroughly cleaned and finely ground at the maximum speed with a Moulinex mixer for 3 min (Type 716, France), to pass through a 1 mm^2^ sieve. Then, the powder was defatted with n-hexane for 8 h, and the defatted meal was dried and stored at 4°C till analysis.

Lupin protein isolate was prepared from defatted lupin flour by acid–base precipitation as described by Johnson and Brekke (1983). Defatted lupin flour was dispersed in water at a concentration of 5% (w/v). Then, the pH was corrected to 9 using 0.1 N NaOH at room temperature before the mixture was agitated for 1 h and centrifuged for 15 min at 2,000 × g. The residue was subjected to a second round of extraction and centrifugation to boost the yield. Protein was precipitated by mixing the extracts and adjusting the pH to 4.5 with 1 N HCl and centrifuged at 2,000 × g for 15 min, followed by decantation of the supernatant and extraction of the proteins. After dialysis overnight and lyophilization, crude protein was dispersed in a small volume of distilled water at pH 7.5.

The 11S globulin was isolated from a defatted meal of lupine seeds following the procedure outlined by Abdel-Shafi et al. (2019a). To isolate the soluble components from the defatted lupin seed meal, 10 g were dissolved in 150 ml of buffer [0.03 mol/L Tris HCl at pH 8.5, 0.4 M NaCl, 10 mM mercaptoethanol, 1 mM EDTA, and 0.02% (w/v) NaN_3_] and centrifuged at 5,000 × g for 10 min. The solution was agitated for 1 h in a water bath at 45°C. Then, ammonium sulfate (65%−85%) precipitated 11S globulin. Following dispersing and solubilizing the precipitate, the salts were removed from the solution by dialysis using the same buffer for 48 h.

#### 2.1.2 . Esterification of 11S globulin

The procedure mentioned by Sitohy et al. (2000, 2001) was employed to esterify lupine seed 11S globulin. Esterification of 11S globulin was performed by dispersing the protein in more than 99.5% pure methanol at a concentration of 5% by weight. Hydrochloric acid was injected dropwise at the beginning of the process at a sufficient concentration to induce the protonation of carboxylates at a 50-molar ratio (mole acid/mol carboxyl group). For 10 h, we stirred each reaction mixture at 4°C. The samples were centrifuged at 10,000 × g for 10 min, following the completion of the 10 h reaction time. The resulting precipitate was dialyzed for 3 days at 4°C against distilled water and then lyophilized after being dissolved in the necessary volume of distilled water at pH 7.5. The extent of esterification of proteins was quantified according to Bertrand-Harb et al. (1991).

#### 2.1.3 . Chemical characterization

Protein pH–solubility curves were assayed using lyophilized 11S globulins and M11S from lupine seeds in the pH range of 2–10 (Chobert et al., 1991). Urea-PAGE of 11S globulins and M11S was performed following the procedure by Osman et al. (2014).

The potassium bromide (KBr) pellet method (Souillac et al., 2002) estimated the infrared spectra of protein samples at 25°C using an FT-IR spectrometer (Nicolet Nexus 470, DTGS, Thermo Scientific, Waltham, MS, USA). The ^1^H NMR spectra were assayed on a Bruker Avance III 400 MHZ High-Performance Digital FT-NMR spectrometer using dimethyl sulfoxide (DMSO-d6) as the solvent. Tetramethylsilane (TMS) was used as an internal standard to report the chemical shifts in δ (ppm).

### 2.2 . Antibacterial activities against gram-negative and gram-positive bacteria

The antibacterial activities of lupine seed 11S and M11S were bio-assayed against seven tested pathogenic bacteria through disk diffusion assay (Abdel-Hamid et al., 2016) and agar well diffusion assay (Abdel-Shafi et al., 2019b).

#### 2.2.1 . Minimum inhibitory concentration

Minimum inhibitory concentration (MIC) was defined using agar well diffusion and disk diffusion methods. The MIC of an antimicrobial agent was taken as the lowest concentration (μg/ml) inhibiting the visible growth of a microorganism after overnight incubation.

#### 2.2.2 . Agar well diffusion assay

The conventional well diffusion assay was used to determine the antibacterial activity against 11S globulin and the M11S against seven tested bacteria (Nanda and Saravanan, 2009). Pure cultures of the bacterial strains were subcultured on brain heart infusion broth (BHIB) at 37°C on a rotary shaker at 200 rpm. The exponential phase culture of each strain was adjusted to 1.05 × 10^9^ CFU ml^−1^ before spreading uniformly onto individual plates using sterile cotton swabs. Wells of 6 mm in diameter were created on BHI agar employing a gel-puncturing tool. Aliquots (40 μl) of tested 11S solutions (0, 0.1, 0.3, 0.5, 1, 2, 4, and 8 μg/ml) and M11S solutions (0, 0.0125, 0.025, 0.05, 0.1, 0.3, and 0.5 μg/ml) were transferred into each well. A transparent ruler measured the developed inhibition zone diameters after 24-h incubation at 37°C.

#### 2.2.3 . Disk diffusion assay

The antibacterial activities of 11S and the M11S globulins were also tested against the seven experimental pathogenic bacteria utilizing the Kirby–Bauer disk diffusion method (Hudzicki, 2009). Bacterial suspensions were evenly spread over the surfaces of nutrient agar plates. Then, sterilized 6-mm paper disks were soaked in different dilutions of 11S globulin (0, 0.1, 0.3, 0.5, 1, 2, 4, and 8 μg/ml) and various dilutions of M11S globulin (0, 0.0125, 0.025, 0.05, 0.1, 0.3, and 0.5 μg/ml) and placed on the top of nutrient agar medium allowing appropriate distances separating the samples. The agar plates were incubated at 37°C for 24 h before the diameters of inhibition zones (mm) were measured using a millimeter ruler. Finally, the diameter of the original disks (6 mm) was subtracted from the total zone diameters, giving the net inhibition zone.

#### 2.2.4 . Comparison of MICs of lupine 11S and M11S with specific antibiotics

The antibiotic sensitivity test was carried out on seven tested pathogenic bacteria using four antibiotics (Abdel-Shafi et al., 2016). The MICs of antibiotics, such as chloramphenicol, gentamycin, ciprofloxacin, ciprofloxacin, vancomycin, ciprofloxacin, and ciprofloxacin antibiotics, were determined against *Listeria ivanovii, Klebsiella oxytoca, Salmonella typhimurium, Listeria monocytogenes, S. aureus, P. aeruginosa*, and *Proteus mirabilis*, respectively.

#### 2.2.5 . Bacterial growth curve (turbidity test)

An aliquot (50 μl) of 1 MIC of each tested substance (11S and M11S) was added to 4-h-old bacterial cultures in BHIB, incubated at 37°C, and dispensed into the wells of a 96-well plate. The negative control was BHIB only, whereas the positive control contained cell cultures without adding treatment. Growth was determined by measuring turbidity (optical density) at 600 nm for 24 h using a microplate reader (Bio-Rad 680XR, Hertfordshire, U.K.).

#### 2.2.6 . Biofilm inhibition activity

The anti-biofilm activity of 11S and M11S was assessed according to the protocol mentioned by Saporito et al. (2018). In brief, overnight cultures of the seven tested bacteria were 100 times diluted before inoculating 90 μl of bacterial suspension into wells prefilled with 10 μl of the tested substances at concentrations equivalent to 1 MIC, 0.1 MIC, and 0.01 MIC in a microtiter plate. In the control wells, 10 μl of MQ-water was added instead of the sample. After 24-h incubation at 37°C, the supernatants were removed, and the wells were gently washed twice with 150 μl/well of phosphate-buffered saline (PBS) to remove non-adhered planktonic bacteria and cellular debris. Next, the attached biofilms were stained by adding 125 μl/well of crystal violet (0.1% w/v in water) and incubated for 10 min at room temperature. Then, the excess dye was washed out with PBS while the stained biofilm was dissolved by adding 200 μl ethanol (96%) to each well for 10 min. Eventually, 100 μl of each well was transferred to a clean flat bottom microtiter plate, and the absorbance was recorded at 595 nm in a microplate reader (Synergy HT, BioTek). The biofilm inhibition (%) was estimated by comparing the optical density values for the treated samples with the untreated control according to the following formula:


$$\begin{array}{l} \text{Biofilm inhibition }(\%)=[(\text{OD595 control}-\text{OD595 sample})\\ \text{ / OD595 control}]\;\times \text{ 100}. \end{array}$$

#### 2.2.7 . Minimum biofilm inhibitory concentration

Inhibition of biofilm formation was assessed according to Nostro et al. (2007). Aliquots (100 μl) of an overnight culture (108 CFU ml^−1^) in BHIB supplemented with 1% (w/v) glucose were dispensed into each well of a 96-well plate and combined with another 100 μl of 11S (0.05, 0.1, 0.3, 0.5, 1, 2, and 4 μg/ml) or 100 μl of M11S (0.006, 0.0125, 0.05, 0.1, 0.3, and 0.5 μg/ml) for M11S. The negative control was BHIB only, whereas the positive control contained cell cultures without 11S or M11S. Following 24-h incubation at 37°C, the liquid layer of each well was decanted and gently rinsed twice with 300 μl of PBS (pH: 73 ± 03). The plates were air-dried for 30 min, stained with 0.1% (w/v) crystal violet for 30 min at room temperature (Wijman et al., 2007), washed three times with PBS (200 μl per well), and dried. The crystal violet was, then, solubilized using 10% (v/v) glacial acetic acid, and the OD was measured at 595 nm using a microplate reader (Bio-Rad 680XR). The minimum biofilm inhibitory concentration (MBIC) was defined as the lowest level of the tested substance causing at least 90% inhibition in biofilm biomass compared with the untreated control using the following formula:


$$\begin{array}{l} \text{Biofilm inhibition }\left(\%\right)=\left[1-\left(\text{OD test }/\;\text{OD control}\right)\right]\;\times \;100. \end{array}$$

#### 2.2.8 . Quantitative biofilm formation assay

The quantitative biofilm formation by both *S. aureus* and *K. oxytoca* was assayed using the protocol according to Adukwu et al. (2012). Clean, grease-free glass slides were placed in 100 ml screw-cap bottles containing 48 ml of BHIB and autoclaved. Then, the medium was inoculated with 2 ml of cultures grown in BHIB, combined with 10 μl of 11S and M11S at concentrations equivalent to MBIC, and incubated for 24 h at 37°C under shaking conditions at 150 rpm. The glass slides were aseptically removed and washed in sterile PBS to remove unattached cells. The cells were removed by rubbing with a sterile cotton swab (Hi-Media). The swab was then transferred to 10 ml PBS and shaken vigorously, and serial 10-fold dilutions of each strain were plated on BHI agar. Multiple swabs were used for the same area and inoculated in PBS to limit the variations in the data resulting in the incomplete removal of the cells from the glass slides. Colony count was performed and calculated for cells in biofilm/cm^2^. The experiment was repeated thrice.

#### 2.2.9 . Scanning electron microscopy of bacterial biofilm

Two bacteria, *S. aureus* (gram-positive) and *K. oxytoca* (gram-negative), were selected to compare the morphological appearance of the bacterial biofilm developed in the absence or presence of 11S and M11S using Jeol JSM-6510 L.V. Scanning electron microscopy (SEM) and following the standard protocols (Adukwu et al., 2012). Two-cm diameter sterile stainless-steel disks (Goodfellow Cambridge Ltd, Huntingdon, U.K.) were immersed in the wells of six-well plates (Nunclon Surface, Roskilde, Denmark) containing each 5 ml of BHIB and 100 μl of an overnight culture (108 CFU ml^−1^). Then, the two tested bacteria were treated with 1 MIC of 11S globulin and M11S, i.e., 0.5 and 0.025 μg/ml in the case of *S. aureus* and 0.5 and 0.1 μg/ml in the case of *K. oxytoca*, respectively. The control for each bacterium did not receive any treatment. All plates were placed in a shaking incubator for 24 h of incubation period, after which the disks were removed and gently washed three times with sterile PBS to eliminate the loosely attached bacterial cells, followed by a 2-h fixation with 2.5% (v/v) glutaraldehyde in PBS solution at 4°C. Finally, the plates were washed twice with PBS for 10 min before dehydration with graded ethanol concentrations: 30%, 50%, 70%, 90%, and 100% (v/v), followed by a 10-min subjection to serial dilutions of acetone (30%, 50%, and 100%) at 25°C. The samples were then dried to a critical point using an automated critical point dryer (Leica EM CPD300, GmbH, Mannheim, Germany), before coating with gold–palladium and observation at 25 and 30 kV.

#### 2.2.10 . Statistical analysis

SPSS program version 23 was used to statistically analyze the mean and standard deviation data. Two-way ANOVA tests were performed to compare the different microorganisms (i.e., *L. monocytogenes, K. oxytoca, L. ivanovii, S. aureus, P. mirabilis, P. aeruginosa, and S. typhimurium*), as well as the different concentrations, (i.e., 0.05, 0.1, 0.3, 0.5, 1, 2, 4, and 8 μg/ml for 11S and 0.0125, 0.025, 0.05, 0.1, 0.3, and 0.5 μg/ml for M11S), and the interactions between them for inhibition zone diameter. The null hypothesis was rejected if the ANOVA *p*-value was < 0.05, indicating statistically significant differences among the group means. When the *p*-value was ≥0.05, the null hypothesis was not rejected, indicating that no statistically significant differences were detected. The two-way ANOVA test with a *post-hoc* test using Duncan's test was applied to make multiple comparisons between the averages of different groups. Means followed by the same letter were not significantly different at the 5% probability level (Duncan's multiple range tests). The results were presented as the means of three replicates ± SD. The statistical analyses which were conducted using two-way ANOVA and Duncan's multiple-range test accurately reflect the methods applied to the original datasets.

## 3 . Results

### 3.1 . Chemical characterization of 11S and M11S

**Figure 1:**
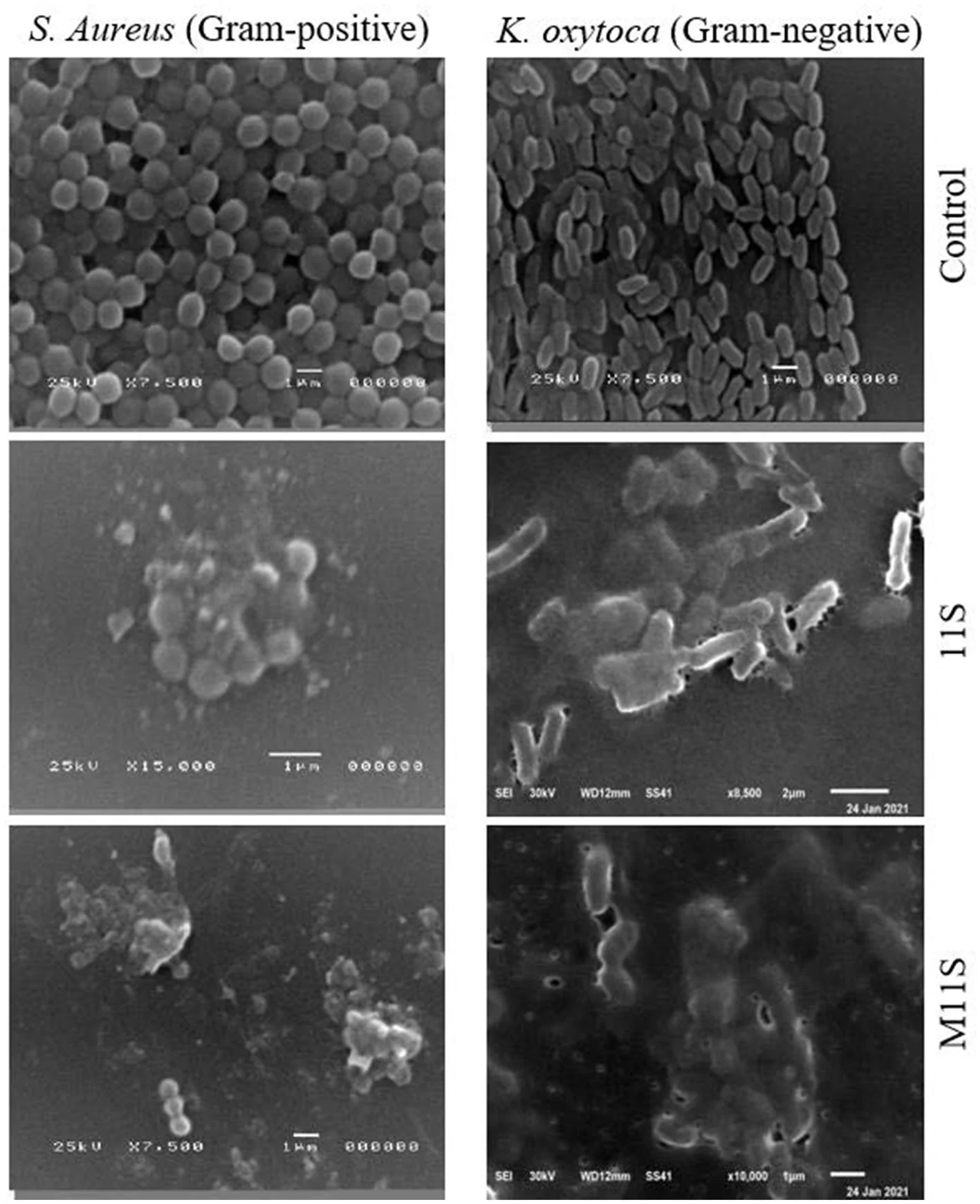
**(A)** UREA-PAGE electropherogram and pH–solubility curves of native (11S) and methylated lupine seed globulin (M11S), **(B)** FT-IR spectra of lupine seed 11S and its methylated derivative M11S globulins, and **(C)** 1HNMR spectroscopy of native (11S) and its methylated 11S globulin (M11S).

The data in Figure 1A confirm that esterifying lupine 11S globulins accelerated its migration into the cathode in Urea-PAGE. This change was further confirmed by tracing the pH–solubility curves of both the native (11S) and esterified protein (M11S), indicating that the isoelectric point of the methylated form (M11S) was relatively higher (pH 8.8) than that of the native form (11S), i.e., pH 7.3.

**Figure 2:**
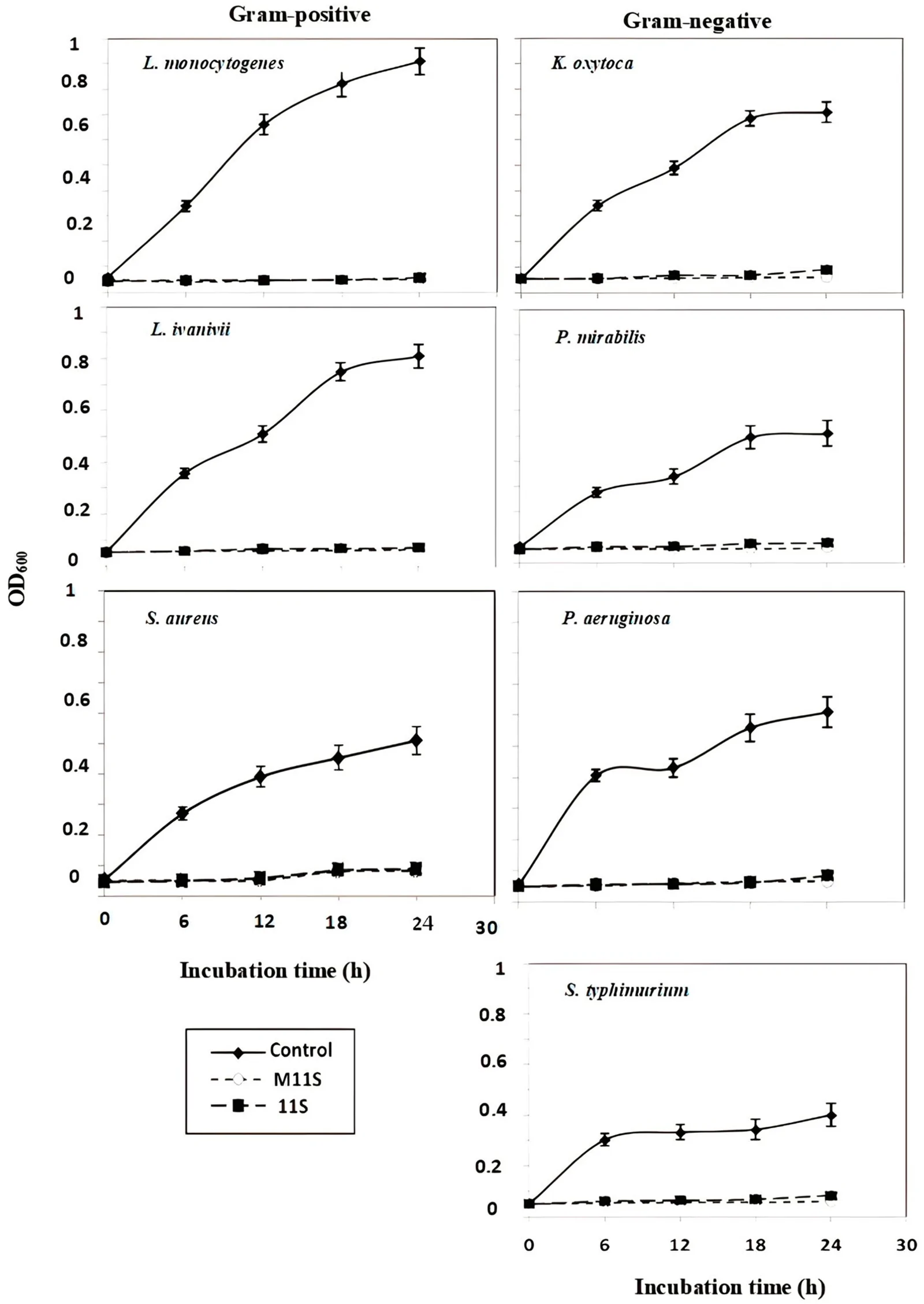
Twenty-four-hour growth curves of seven pathogenic bacteria (three gram-positive and four gram-negative) in the presence of 1.0 MIC of 11S and M11S compared with control (untreated bacteria). All the values were the means of three replicates ± SD.

The FTIR data in Figure 1B show that the modified lupine globulin (M11S) contains three specific absorption peaks at 3,067, 1716, and 1,172 cm^−1^, which are absent in the native form (11S), referring to the presence of aliphatic CH_3_ group, C=O stretching, and C=O stretching ester, respectively, according to I.R. Spectrum Table (sigmaaldrich.com). Alternatively, the native form (11S) showed an I.R. absorption peak at 1,391 cm^−1^, absent from the modified form (M11S), referring to the free carboxylate groups. ^1^H NMR of native 11S globulin indicates the presence of main six δ signals at 1.51 (s, 1H, CH_3_), 5.09 (s, 2H, OCH_2_), 5.52 (s, 1H, Ar-H), 7.48 (d, 1H, *J* = 6.8 Hz, Ar-H), 7.50 (s, 1H, N.H.), and 7.58–7.66 (m, 3H, Ar-H). The esterified product (M11S) indicated other 6 signals, 4 of them are shared with the native form (11S), while the other 2 are different (Figure 1C), precisely the signal 5.09 which is present in the native structure (11S) and the signal 2.68 which is present only in the modified state (M11S). Additionally, the signal appearing at 7.5 Hz in the native protein (11S) shifted to 8.56 Hz in the modified protein (M11S).

### 3.2 . Antibacterial activity of 11S and M11S

Initially, the antibacterial activities of 11S and M11S were tested at a high concentration (1000 μg/ml) by the disk diffusion method, demonstrating antibacterial activity against all the tested bacteria, while the whole lupine seed protein isolate (LPI) did not produce any inhibition zones (data not shown).

#### 3.2.1 . Minimum inhibitory concentration

The data in Table 1 and Supplementary Figure 1 show that lupine 11S affected the gram-positive and gram-negative bacteria (*S. aureus and K. oxytoca*, respectively) equally in terms of the specific MIC, detected at 0.5 μg/ml for both. However, the inhibition zone area data indicated that the gram-positive bacteria were more susceptible than the gram-negative ones. Chemically modifying 11S into the methylated form (M11S) enhanced the antibacterial action, where the effect on the gram-positive bacteria was much more pronounced than on the gram-negative bacteria. Regarding MIC, M11S recorded 0.025 and 0.100 μg/ml against the gram-positive and the gram-negative bacteria, respectively. Expanding the analysis to the whole bacterial collection (3 gram-positive and 4 gram-negative) confirmed that 11S and M11S were more inhibitory on gram-positive than gram-negative bacteria (Table 1). At the same time, the antibacterial action of M11S was more effective on the two bacterial types (gram-positive and gram-negative bacteria) than on 11S. M11S achieved the lowest MIC against both gram-positive and gram-negative bacteria, i.e., 0.025, 0.025, and 0.1 μg/ml in the first case (*L. monocytogenes, S. aureus, and L. ivanovii*), respectively, and 0.1, 0.3, 0.5, and 0.1 μg/ml in the second case (*K. oxytoca, P. mirabilis, P. aeruginosa, and S. typhimurium*), respectively. The MIC of M11S was 4, 20, and 10 times less than the corresponding values of 11S against the three tested gram-positive bacteria, respectively, and 5, 3, 8, and 20 times lower than the corresponding values of 11S against the gram-negative bacteria, respectively.

**Table 1:** Bacterial inhibitory action of graded concentrations of lupines 11S and M11S using agar well diffusion assay as displayed by the Inhibition zone diameter (mm /11S μg ml^−1^).

Microorganism	Lupine 11S globulin (μg/ml)								Microorganism effect
	0.05	0.1	0.3	0.5	1	2	4	8	
	**Inhibition zone diameter (mm)**								
Gram-positive									
*Listeria monocytogenes*	0q ± 0	16o ± 0	28 ± 0.58	30i ± 0	29.33j ± 0.33	33g ± 1	46c ± 0.58	49a ± 1	28.917a ± 3.06
*Staphylococcus aureus*	0q ± 0	0q ± 0	0q ± 0	15.30o ± 1.2	24n ± 0	26m ± 1.15	30.33ij ± 0.67	39.66e ± 0.33	16.92c ± 3.04
*Listeria ivanovii*	0q ± 0	0q ± 0	0q ± 0	0q ± 0	13.00pn ± 0.58	29.33j ± 0.88	36f ± 0.58	44.33d ± 0.1	15.33d ± 3.62
Gram-negative									
*Klebsiella oxytoca*	0q ± 0	0q ± 0	0q ± 0	31hi ± 0.58	33.00g ± 1.73	47b ± 0	48ab ± 0.58	49a ± 0	26.00b ± 4.4
*Proteus mirabilis*	0q ± 0	0q ± 0	0q ± 0	0q ± 0	0.00q ± 0	28l ± 0.58	30ijk ± 0.58	32gh ± 0.58	11.25e ± 3.04
*Pseudomonas aeruginosa*	0q ± 0	0q ± 0	0q ± 0	0q ± 0	0.00q ± 0	0q ± 0	28.66kl ± 0.67	31hi ± 0	7.46g ± 2.7
*Salmonella typhimurium*	0q ± 0	0q ± 0	0q ± 0	0q ± 0	0.00q ± 0	24.00n ± 0.58	25mn ± 0	32.ghh ± 1.15	10.12f ± 2.77
Concentration effect	0h ± 0	2.28g ± 1.25	4f ± 2.2	10.9e ± 3.01	14.19d ± 3.04	26.76c ± 2.91	34.85b ± 1.86	39.57a ± 1.68	
	**Lupine M11S globulin (μg/ml)**								
	**0.0125**	**0.025**	**0.05**	**0.1**	**0.3**	**0.5**			
	**Inhibition zone diameter (mm)**								
Gram-positive									
*Listeria monocytogenes*	0.p ± 0	32.00d ± 0	34.00c ± 1	40.00b ± 0	45.00a ± 1	46.00a ± 1			39.40a ± 5.88
*Staphylococcus aureus*	0p ± 0	20.00i ± 1	24.00g ± 0	30.00e ± 1	31.00de ± 1	35.00c ± 0			28.00b ± 5.55
*Listeria ivanovii*	0p ± 0	0.00p ± 0	0.00p ± 0	18.00j ± 1	22.00h ± 1	28.00f ± 1			13.60c ± 11.97
Gram-negative									
*Klebsiella oxytoca*	0p ± 0	0p ± 0	0p ± 0	18.00j ± 0	20i ± 1	28.00f ± 1			13.20c ± 11.69
*Proteus mirabilis*	0p ± 0	0p ± 0	0p ± 0	0p ± 0	14.00l ± 1	16.00k ± 1			6.00e ± 7.65
*Pseudomonas aeruginosa*	0p ± 0	0p ± 0	0p ± 0	0p ± 0	0p ± 0	12m ± 1			2.40f ± 4.98
*Salmonella typhimurium*	0p ±	0p ± 0	0p ± 0	16k ± 1	17jk ± 0	23gh ± 1			11.20d ± 9.80
Concentration effect	0.00 ± 0	7.429e ± 0	8.28d ± 12.5	17.42c ± 13.7	21.28b ± 13.5	26.85a ± 13.3			

Values are the means of three replicates ± SD.

#### 3.2.2 . Inhibition of liquid bacterial growth

The data in Figure 2, representing the 24-h growth curves of seven pathogenic bacteria subjected to one MIC of 11S and M11S, show general substance-based growth inhibition. In the liquid media, 1.0 MIC of both agents was nearly sufficient to completely prevent the 24-h liquid bacterial growth of all the tested microorganisms. *Listeria monocytogenes* and *K. oxytoca* were the most inhibited organisms. The untreated control and 11S growth datasets' curves were intentionally shared between the two publications (Abdel-Shafi et al., 2023 and Enan et al., 2023, retracted) as they originated from the same experimental series, as a common experimental baseline for both. Alternatively, the two treatment groups datasets (BS and M11S) were unique for each of (Abdel-Shafi et al., 2023, retracted and Enan et al., 2023), respectively. Additionally, the scientific objectives differed totally between the two manuscripts.” It should be clarified that the retraction of the first publication (Abdel-Shafi et al., 2023, retracted) has no bearing on the experimental data reused in the present article. The reuse of these shared controls was not disclosed in the original manuscript since the two articles were nearly prepared at the same time.

#### 3.2.3 . Comparison of the MIC of 11s and M11S with specific antibiotics

**Figure 3:**
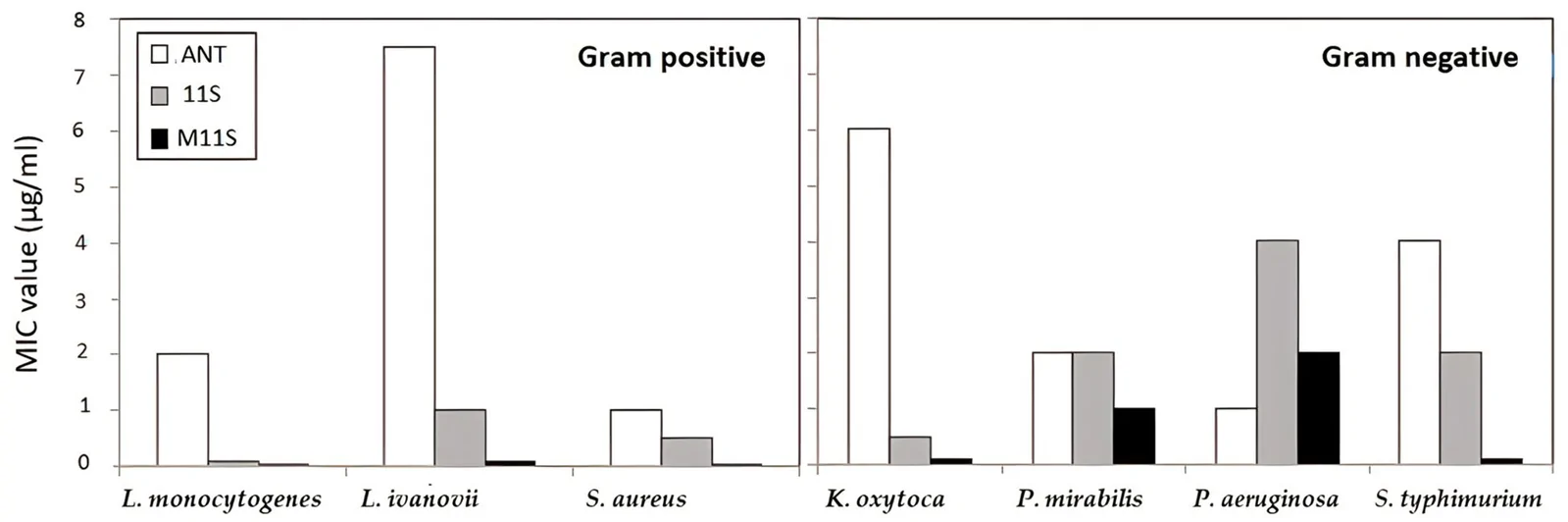
Comparison of MIC of the tested lupine 11S globulins and its methylated form M11S. Chloramphenicol, ciprofloxacin, and vancomycin were used as specific against *Listeria ivanovii, Listeria monocytogenes*, and *Staphylococcus aureus*, (MIC at 7.5, 2, and 1 μg/ml, respectively). The four investigated gram-negative bacteria (*Klebsiella oxytoca, Salmonella typhimurium, Pseudomonas aeruginosa*, and *Proteus mirabilis*) were specifically susceptible to gentamycin, ciprofloxacin, ciprofloxacin, and ciprofloxacin at MIC 6, 4, 1, and 2 μg/ml, respectively.

Figure 3 presents comparative measurements of MIC of the 11S and M11S against seven pathogenic bacteria as compared with the action of specific antibiotics. The antibiotics such as chloramphenicol, ciprofloxacin, and vancomycin were considered as typically specific against the three investigated gram-positive bacteria, namely *L. monocytogenes, L. ivanovii, and S. aureus*, exhibiting MIC at 2, 7.5, and 1 μg/ml, respectively, while the four investigated gram-negative bacteria were explicitly susceptible to gentamycin and ciprofloxacin. The first bacterium (*K. oxytoca*) showed MIC at 6 μg/ml. In contrast, the three other bacteria (*P. mirabilis, P. aeruginosa*, and *S. typhimurium*) were susceptible to ciprofloxacin at 2, 1, and 4 μg/ml, respectively. Comparatively, the MICs of M11S were much lower than the specific antibiotics but slightly lower than 11S.

In the case of the three tested gram-positive bacteria, the MICs of 11S and M11S were 0.1 and 0.025 μg/ml against *L. monocytogenes* while 1 and 0.1 μg/ml against *L. ivanovii*, respectively. Compared with the specific antibiotics, the MICs of M11S were 80, 75, and 40 times lower than those of the specific antibiotics against *L. monocytogenes, L. ivanovii*, and *S. aureus*, respectively. At the same time, they were 60, 2, 0.5, and 40 times lower than the respective values against the gram-negative bacteria, such as *K. oxytoca, P. mirabilis, P. aeruginosa*, and *S. typhimurium*.

#### 3.2.4 . Anti-biofilm activity of 11S and M11S

**Figure 4:**
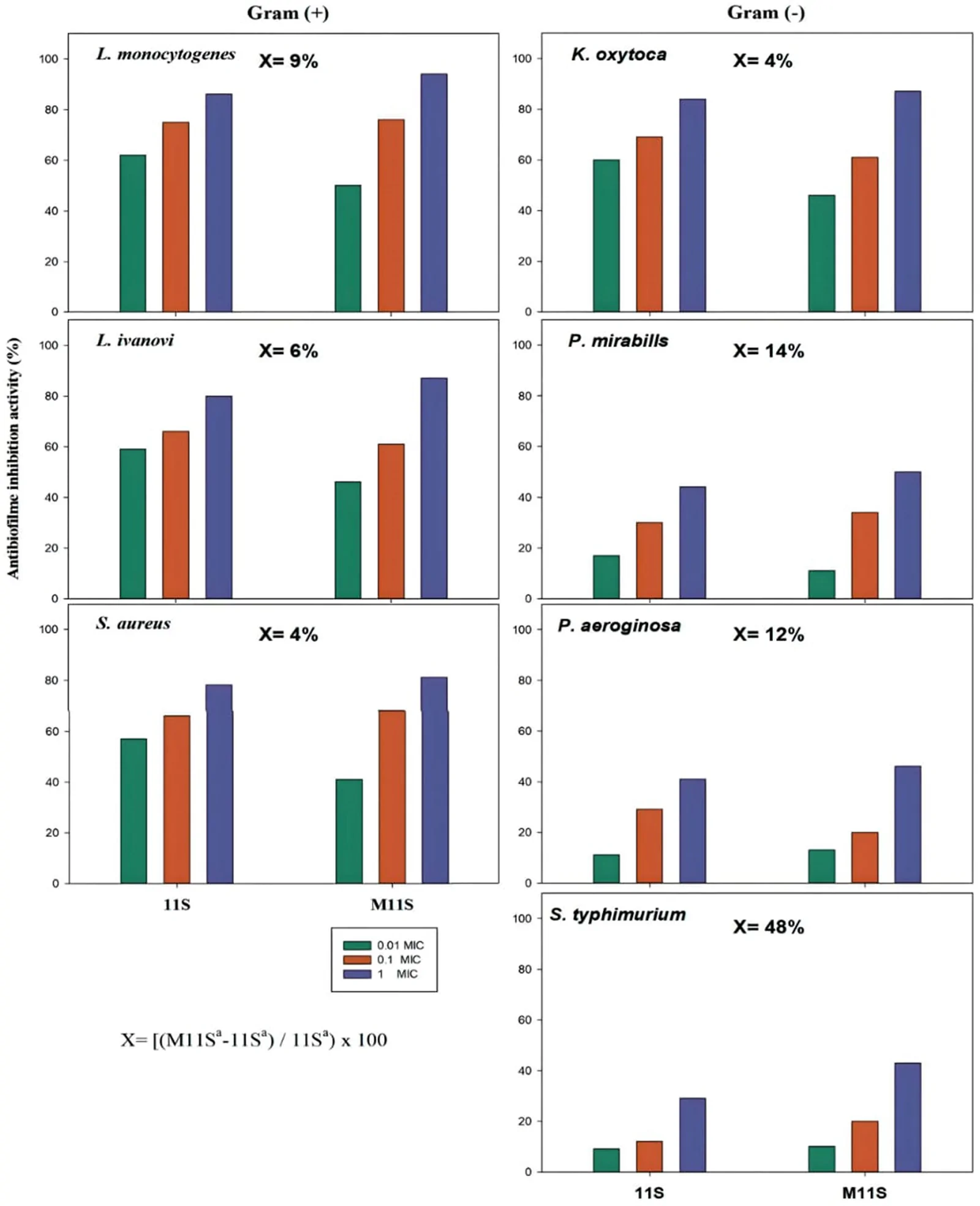
Inhibitory activity of native (11S) and methylated (M11S) lupine seed globulin on the bacterial biofilm formation by gram-positive and gram-negative bacteria at 0.01, 0.1, and 1.0 MIC levels. The increase in anti-biofilm activity (*x*) of M11S over 11S was calculated at 1 MIC according to the equation under the figure, where *a* is the anti-biofilm activity.

The data in Figure 4 show that the inhibitory action of 11S and M11S applied at 0.01, 0.1, and 1.0 MIC levels on the bacterial biofilm formation was considerably concentration-dependent. One MIC of 11S and M11S inhibited the gram-positive film formation by 80–86% and 85–94%, respectively. Lower anti-biofilm inhibitory actions occurred against the gram-negative bacteria, amounting to 29%−44% and 43%−50% by 11S and M11S (1 MIC), respectively. M11S achieved 9%, 6%, and 4% increases in the biofilm inhibitory action in the case of the gram-positive bacteria (*L. monocytogenes, L. ivanovii*, and *S. aureus*). The anti-biofilm formation activity exerted by M11S against the gram-negative bacteria such as *K. oxytoca, P. aeruginosa, P. mirabilis*, and *S. typhimurium* registering 4%, 14%, 12%, and 48% increases over 11S.

#### 3.2.5 . Minimum biofilm inhibitory concentration

Matching the inhibitory action of 11S and M11S with bacterial film formation by gram-positive and gram-negative bacteria in terms of the minimum biofilm inhibitory concentration (MBIC), with their corresponding MICs (Table 2), revealed close low values for each. Lupine 11S globulin prevented biofilm formation by gram-positive bacteria (*L. monocytogenes, S. aureus*, and *L. ivanovii*) at MBIC of 0.1, 0.5, and 1 μg/ml against 0.025, 0.025, and 0.1 μg/ml for M11S. Similarly, the MBIC against the gram-negative bacteria, such as *K. oxytoca, P. mirabilis, P. aeruginosa*, and *S. typhimurium*, recorded 0.5, 2, 4, and 2 μg/ml in the case of 11S against 0.1, 0.3, 0.5, and 0.1 μg/ml in the case of M11S. The MBIC and MIC of 11S and M11S (Table 2) revealed very close values.

**Table 2:** Minimum biofilm inhibitory concentration (MBIC) of 11S and M11S assayed by microtiter plate technique as compared to the minimum inhibitory concentration (MIC).

Bacterial type	Microorganism	11S		M11S		MBIC M11S/MBIC 11S (%)
		MIC (μg/ml)	MBIC (μg/ml)	MIC (μg/ml)	MBIC (μg/ml)	
Gram-positive	*Listeria monocytogenes*	0.1	0.1	0.025	0.025	25
	*Staphylococcus aureus*	0.5	0.5	0.025	0.025	5
	*Listeria ivanovii*	1	2	0.1	0.3	15
Gram-negative	*Klebsiella oxytoca*	0.5	0.5	0.1	0.1	20
	*Proteus mirabilis*	2	2	0.3	0.5	25
	*Pseudomonas aeruginosa*	4	2	0.5	0.5	25
	*Salmonella typhimurium*	2	2	0.1	0.3	15

MIC, the lowest concentration inhibiting the visible bacterial growth; MBIC, the lowest concentration causing at least 90% inhibition in biofilm biomass.

#### 3.2.6 . Quantitative biofilm formation assay

The quantitative biofilm formation assay was conducted to enumerate both *S. aureus* and *K. oxytoca* after overnight growth on BHIB in the absence or presence of 11S and M11S at 1 MBIC at 37°C under 150 rpm shaking condition followed by 24 h of incubation. Colony count was performed and calculated as cells per 1 cm^2^ biofilm. The data in Supplementary Figure 2 revealed the actual number of cells present in the biofilms formed by *S. aureus* (gram-positive) and *K. oxytoca* (gram-negative) as 7.23 and 6.24 log CFU/cm^2^ in the absence of the two antibacterial agents. Both 11S and M11S, at 1 MBIC at 37°C under 150 rpm shaking condition followed by 24-h incubation, revealed no attached cells, confirming that they have equal potencies to completely counteract the formation of microbial biofilms developed by either gram-positive or gram-negative bacteria. This test indicated complete prevention of the biofilm formation in each case under low concentrations of the two antibacterial agents, i.e., 0.5 and 0.5 μg/ml in the case of 11S and 0.025 and 0.1 μg/ml in the case of M11S against *S. aureus* and *K. oxytoca*, respectively.

#### 3.2.7 . SEM image analysis

**Figure 5:**
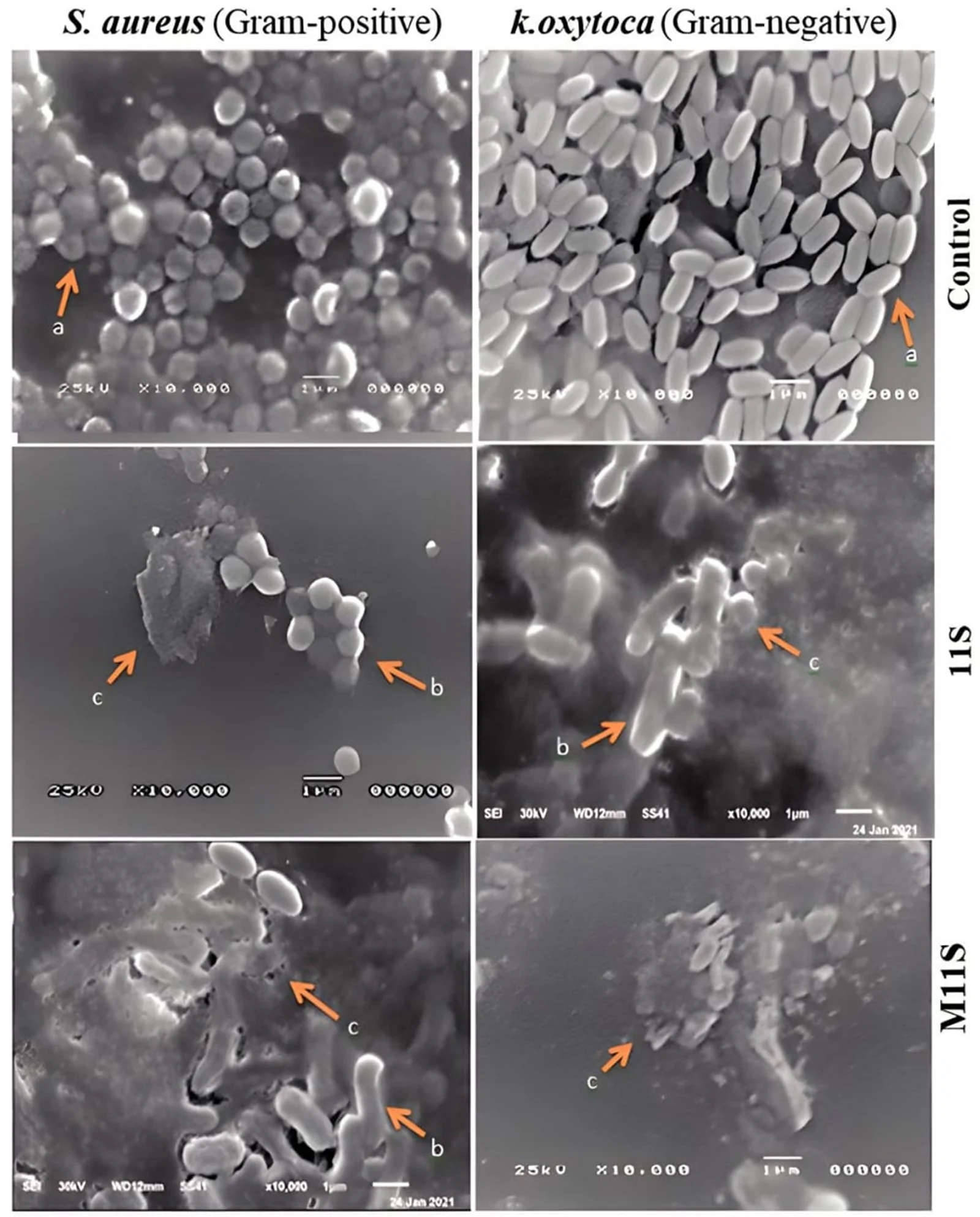
Scanning electron micrographs of biofilm formation by *Staphylococcus aureus* (Gram-positive) and *Klebsiella oxytoca* (Gram-negative) treated with 1 MBIC of native 11S globulin or methylated 11S (M11S). Control cells formed adherent, confluent biofilms; 11S treatment caused moderate biofilm disruption; M11S treatment resulted in major loss of confluence and extensive cellular damage. For *S. aureus*, the untreated control image was acquired at 25 kV, × 7,500 magnification with a 1 μm scale bar; the 11S-treated sample (0.5 μg/ml) was imaged at 25 kV, × 15,000 with a 1 μm scale bar; and the M11S-treated sample (0.025 μg/ml) was imaged at 25 kV, × 7,500 with a 1 μm scale bar. For *K. oxytoca*, the untreated control image was acquired at 25 kV, × 7,500 with a 1 μm scale bar; the 11S-treated sample (0.5 μg/ml) was imaged at 30 kV, × 8,500 with a 2 μm scale bar; and the M11S-treated sample (0.1 μg/ml) was imaged at 30 kV, × 10,000 with a 1 μm scale bar.

The SEM images in Figure 5 represent the bacterial biofilms formed by two bacteria, namely S. *aureus* (gram-positive) and *K. oxytoca* (gram-negative), on stainless-steel disks immersed in the wells of 6-well plates containing BHIB and 100 μl of an overnight culture (10^8^ CFU ml^−1^) in the absence or presence of 11S and M11S (1 MIC) after 24-h incubation at 37°C. The SEM image of the control *S. aureus* (gram-positive) formed biofilm presents spherical bacterial cells dispersed on the adhered biofilm matrix. The bacterial cells lay on the extracellular polymer surface without fragmented or indented cells. The cells seem adherent to each other and confluent. The presence of 1.0 MBIC concentrations of 11S globulin (0.5 μg/ml) and M11S (0.025μg/ml) in BHIB media containing *S. aureus* and *K. oxytoca* reduced the relative content of the intact cells, thus destroying the biofilm after 24 h of incubation at 37 °C. After treatment with 11S or M11S (1 MBIC), a few regular cells appeared, while most cells were either moderately or entirely deformed. The resulting apparent biofilm deformation was more observed in the case of M11S.

## 4 . Discussion

The enhanced migration of the methylated form (M11S) toward the cathode proves its bigger net positive charges than the native globulin (11S), apparently due to the neutralization of the side-chain negative carboxylate groups on the protein molecule following the study by Mahgoub et al. (2011). Elevating the isoelectric points confirms the more basic nature and higher positive charge of the modified protein, agreeing with Sitohy and Osman (2010). The value of the isoelectric point (IEP) of lupine 11S is higher than other previously studied similar fractions isolated from legumes, e.g., soy protein (Osman et al., 2016) and chickpea protein (Sitohy et al., 2011), explaining the difference in the antibacterial activity according to legume sources. Consequently, the IEP of the methylated (M11S) excelled over the corresponding methylated fractions from other legume sources (Sitohy et al., 2021).

Comparing the FTIR spectrum data of M11S with the native form (11S) confirmed the formation of the ester groups and the disappearance of the free carboxylic groups on the modified protein molecules. The similarity of the other absorption peaks indicates that the modification reaction was mainly restricted to the regions rich in free carboxylic groups arising primarily from residual aspartate and glutamate groups on the surface protein molecules.

The presence of two δ signals in the HNMR profile of M11S (2.68) is different from those of 11S (5.09), whereas the other four signals are similar, confirming that the esterification reaction induced only a few structural changes while maintaining most of the original structural features. Additionally, the shift of the signal appearing at 7.5 Hz in the native protein (11S) referring to the imine groups (s, 1H, N.H.) to be at 8.56 Hz in the modified protein (M11S) may be due to the incorporated electron-donating methyl groups which enhance the electron intensity around the imine groups transforming them into a de-shielded status. Alternatively, the lower levels of proton resonance for most groups in the modified protein (M11S) may refer to a general shielding process by the incorporated methyl groups. Initially, these chemical changes predicted and supported outstanding biological activities of the methylated products as antibacterial and anti-biofilm formation agents.

The values of both MIC and inhibition zones indicated that protein modification generally enhanced the antibacterial activity against the two bacterial types (gram-positive and gram-negative), following similar proteins (Abdel-Shafi et al., 2019b). Both modified plant proteins (Osman et al., 2014) and animal proteins (Chobert et al., 2007) were reported for higher antimicrobial activities. The more effective antibacterial action of M11S than 11S on the two bacterial types (gram-positive and gram-negative bacteria) follows previous results (Sitohy et al., 2013). It can be calculated that the MIC of M11S was 4, 20, and 10 times less than the corresponding values of 11S against the three tested gram-positive bacteria, respectively, and 5, 3, 8, and 20 times lower than the corresponding values of 11S against the gram-negative bacteria, respectively. Thus, M11S can be preferred to 11S as an antibacterial. However, both of them can be classified as broad-specific and highly effective due to their relative richness in hydrophobicity and alkalinity (Sitohy et al., 2012), initiating the electrostatic interactions between the antimicrobial agent's positive charges and the bacterial cell walls or membranes' negatives charges.

The 24-h growth of the tested gram-positive and gram-negative bacteria subjected to one MIC of 11S and M11S was nearly entirely prevented by both agents in agreement with previous results (Osman et al., 2018). However, the antibacterial inhibition recorded in this study seems considerably higher, probably due to some chemical structural differences of the original proteins. The lower levels of MIC of M11S than the specific antibiotic may refer to higher antibacterial action, following previous reports (Sitohy and Osman, 2010). This is the first time to record such minimal MIC values, resulting probably from the specific chemical characteristics of the original globulin (11S) and the esterification process. This enhanced antibacterial activity of M11S is expected to reflect considerable anti-biofilm formation activity.

In the current study, the exhibited high capacity of 11S and M11S to inhibit biofilm formation is evidently due to their antimicrobial effect on the viable bacteria preventing biofilm forming. This association between antimicrobial and anti-biofilm formation was previously observed as treating the surface of a medical appliance with a cationic antimicrobial protein (lysozyme), inhibiting bacterial colonization and preventing biofilm formation (Song et al., 2023).

Alternatively, the cationic M11S and 11S could have affected biofilm formation by disrupting the gel formed by the extracellular polymeric substances. This is supported by Li et al. (2022), which demonstrated that cationic dextrans and polyethyleneimine (PEI) can disrupt *P. aeruginosa* biofilms by inducing a gel-to-sol phase transition of the biofilm matrix. In addition, the cationic conjugates of phthalocyanine derivatives with the nanosphere “NH_2_-ZnONPy” showed great eradication potential on the mixed microbial biofilms of *S. aureus* and *Escherichia coli* and fungus (*Candida albicans*) (Sindelo et al., 2022). The positively charged phthalocyanine analogs showed potential anti-biofilm agents against *S. aureus* and *E. coli* (Openda and Nyokong, 2023). Thus, the current results confirm that the previous studies on the impact of the protein-positive charge on antibacterial efficiency (Abdel-Shafi et al., 2023; Mahmoud et al., 2023) can predict a potential anti-biofilm formation activity.

The observed close values of MBIC and MIC of 11S and M11S suggest that the tested substances have affected the bacteria in their free state before forming the bacterial biofilm. However, the always smaller value of MBIC of M11S than 11S (i.e., only 5%−25%) may refer to a higher anti-biofilm potential. Calculative comparisons substantiated this conclusion as the anti-biofilm inhibitory action of M11S represented 4–20 times that of 11S against the gram-positive bacteria and 4–7 times against the gram-negative bacteria. The recorded lower values of the MBICs of M11S than 11S confirm its higher anti-biofilm capacity, probably due to the higher cationic nature following Sindelo et al. (2022) and Openda and Nyokong (2023).

The quantitative biofilm formation assay of *S. aureus* and *K. oxytoca* revealing no attached cells when subjected to 1 MBIC of 11S and M11S at 37°C indicated equal potencies to completely counteract biofilm formation by low concentrations of the two agents (0.5 and 0.5 μg/ ml). This action resulted probably from a quick adhesion of the tested proteins (11S and M11S) on the surface of Petri dishes due to their cationic nature, preventing the bacterial adhesion, i.e., increased protein adsorption on the solid surface reduced the bacterial adhesion. Since different bacterial proteins are involved in biofilm formation (Abdel-Hamid et al., 2020), the applied agents (11S and M11S) might compete with the bacterial proteins for the adhesion sites on the solid surface, affecting the biofilm formation. The multifunctional soy protein adhesives with antibacterial properties could adhere to solid surfaces (Lei et al., 2022). Accordingly, the positively charged esterified M11S might have electrostatically interacted with the negative charges of the solid glass surface and thus prevented bacterial biofilm formation. Both glass and metallic surfaces have negatively charged surfaces, giving an advantage to the M11S to attach and prevent bacterial biofilm formation. This fact may create different applications of these positively charged proteins in preventing bacterial biofilm formation.

The manifested SEM bacterial ultrastructures revealing a higher degree of damage by M11S than 11S may be due to more vigorous interactions with the bacterial membranes based on its higher net positive charges (Abdel-Shafi et al., 2019b). The evidenced anti-biofilm formation of 11S and M11S against gram-negative (particularly *K. oxytoca*) and gram-positive bacteria (*S. aureus*) is a significant result since the first one is known as an opportunistic and clinically dangerous pathogen (Singh et al., 2011). The second one has multiple public health concerns, is responsible for food poisoning, and can produce several virulence factors (Enan et al., 2020). The SEM-evidenced action of 11S and M11S against the two bacteria followed previously published studies on other similar antibacterials (Mahmoud et al., 2023).

## 5 . Conclusion

Both 11S and M11S lupine seed globulins can be employed as effective antibacterial and anti-biofilm formation agents, both broad-specific and effective. The antibacterial activities of 11S and M11S considerably affected the growth of the pathogenic bacteria either on the solid agar or in liquid media. On the solid agar media, very minimal MIC values marked both 11S (0.1–4.0 μg/ml) and M11S (0,025–0.50 μg/ml), where the MICs of M11S were 4, 20, and 10 times lower than the corresponding values of 11S against the three tested gram-positive bacteria, *L. monocytogenes, S. aureus*, and *L. ivanovii*, respectively, and 5, 3, 8, and 20 times lower than the corresponding values of 11S against the gram-negative bacteria, *K. oxytoca, P. mirabilis, P. aeruginosa*, and *S. typhimurium*, respectively. There was an association between antimicrobial and anti-biofilm formation activities of 11S and M11S as the general antimicrobial effect on viable bacterial cells may prevent biofilm formation. The values of MBIC and MIC of the two substances (11S and M11S) were nearly close, and the MBIC of M11S is always smaller than that of 11S. Both 11S and M11S excelled specific antibiotics in antibacterial efficiency and can be nominated as proper antibacterial substitutes. The more positively charged esterified product (M11S) is highly efficient as an anti-biofilm agent since its more considerable positive charges facilitate its attachment to the negatively charged solid surfaces, either metallic, glass, or plastic, thus preventing bacteria from forming biofilms. Moreover, M11S can be highly recommended as an anti-biofilm agent.

## Data Availability

The original contributions presented in the study are included in the article/Supplementary material, further inquiries can be directed to the corresponding authors.
