# Correction: Controlling bacterial biofilm formation by native and methylated lupine 11S globulins

**DOI:** 10.3389/fmicb.2026.1933458

**Published:** 2026-09-01

**Authors:** Gamal Enan, Seham Abdel-Shafi, Mona El-Nemr, Wesam Shehab, Ali Osman, Mahmoud Sitohy, Basel Sitohy

**Affiliations:** 1 Department of Botany and Microbiology, Faculty of Science, Zagazig University, Zagazig, Egypt; 2 Department of Chemistry, Faculty of Science, Zagazig University, Zagazig, Egypt; 3 Department of Biochemistry, Faculty of Agriculture, Zagazig University, Zagazig, Egypt; 4 Department of Clinical Microbiology, Infection and Immunology, Umeå University, Umeå, Sweden; 5 Department of Radiation Sciences, Oncology, Umeå University, Umeå, Sweden

**Keywords:** 11S globulin, methylated, antibiofilm activity, pathogenic bacteria, FTIR

There was a mistake in Figure 5 as published. Incorrect representative SEM panels were inadvertently incorporated during figure preparation.

The corrected Figure 5, and its caption, appears below:

- Some references were incorrectly transcribed during manuscript preparation, resulting in citation/reference mismatches within the reference list. These errors occurred during manuscript preparation and affected the bibliographic information only.

**Figure 1:**
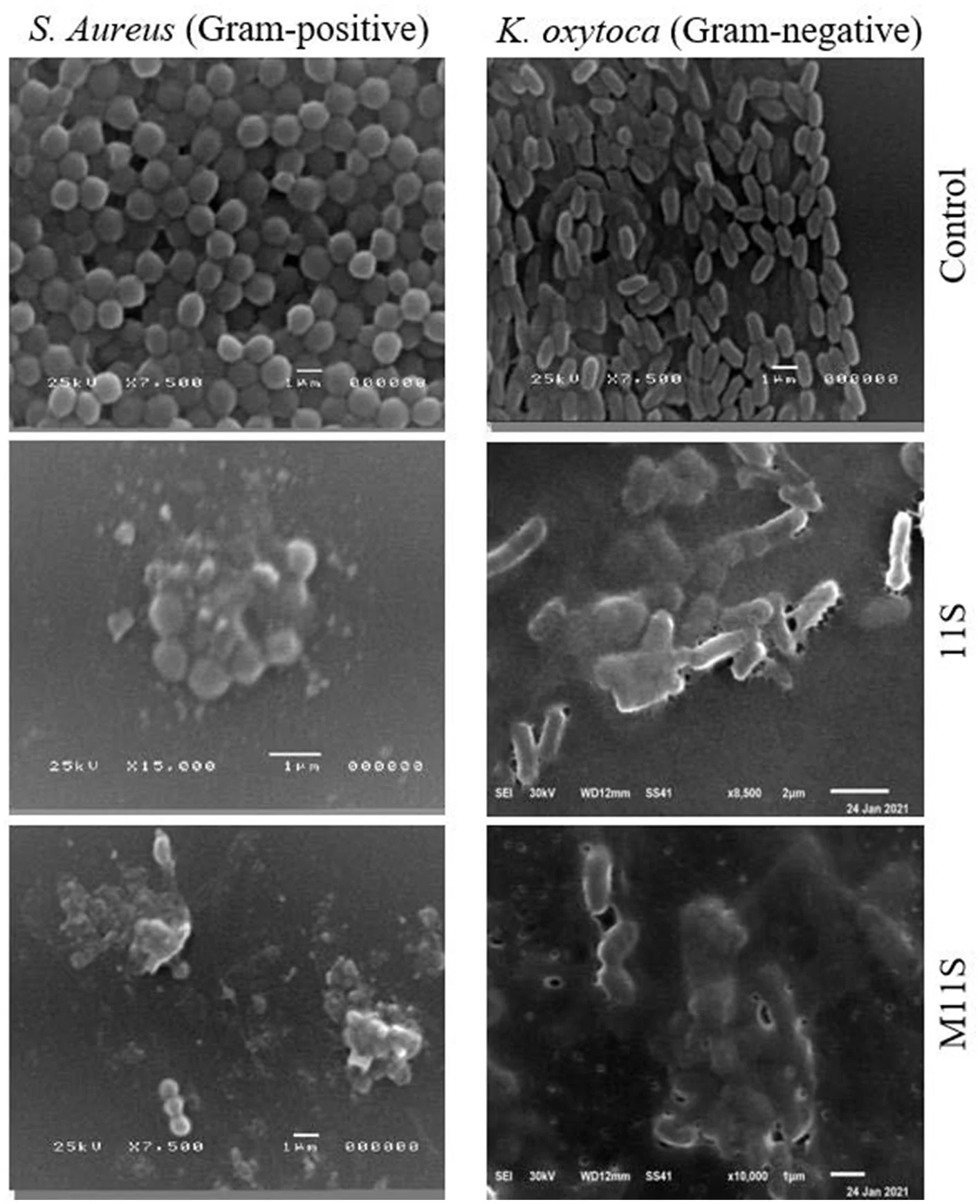
Scanning electron micrographs of biofilm formation by Staphylococcus aureus (Gram-positive) and Klebsiella oxytoca (Gram-negative) treated with 1 MBIC of native 11S globulin or methylated 11S (M11S). Control cells formed adherent, confluent biofilms; 11S treatment caused moderate biofilm disruption; M11S treatment resulted in major loss of confluence and extensive cellular damage. For S. aureus, the untreated control image was acquired at 25 kV, ×7,500 magnification with a 1µm scale bar; the 11S-treated sample (0.5µg/ml) was imaged at 25 kV, ×15,000 with a 1µm scale bar; and the M11S-treated sample (0.025µg/ml) was imaged at 25 kV, ×7,500 with a 1µm scale bar. For K. oxytoca, the untreated control image was acquired at 25 kV, ×7,500 with a 1µm scale bar; the 11S-treated sample (0.5µg/ml) was imaged at 30 kV, ×8,500 with a 2µm scale bar; and the M11S-treated sample (0.1µg/ml) was imaged at 30 kV, ×10,000 with a 1µm scale bar.

The reference “Yarwood, J. M., Bartels, D. J., Volper, E. M., and Greenberg, E. P. (2004). Quorum sensing in *Staphylococcus aureus* biofilms. *J. Bacteriol*. 186, 1838–1850. doi: 10.1128/jb.186.6.1838-1850.2004” has been added to the Reference list.

The citation has now been inserted in the section [**Introduction**, Paragraph 1] and should read: “[Biofilms can protect bacterial species from environmental challenges, e.g., nutrient depletion and the threat of antibiotics (Yarwood et al., 2004)]”

The reference “Pereira, A., Ramos, F., and Sanches Silva, A. (2022). Lupin (*Lupinus albus* L.) seeds: balancing the good and the bad and addressing future challenges. *Molecules* 27:8557. doi: 10.3390/molecules27238557” has been added to the Reference list.

The citation has now been inserted in the section [**Introduction**, Paragraph 2] and should read: “[Lupin globulin consists of two major subunits called α-conglutin (11S and “legumin-like”) and β-conglutin (7S and “vicilin-like”), accounting for ~33 and 45% of the total protein content in Lupinus albus, respectively (Pereira et al., 2022).]”

The reference “Lei, Z., Jiang, K., Chen, Y., Qi, J., Xie, J., Huang, X., et al. (2022). Developing a high strength antibacterial soy protein adhesive by adding a low amount of tetraepoxy L-tyrosine. *Polym. Test*. 112:107643. doi: 10.1016/j.polymertesting.2022.107643” has been added to the Reference list.

The citation has now been inserted in section [4 **Discussion**, Paragraph before last] and should read:

“[The multifunctional soy protein adhesives with antibacterial properties could adhere to solid surfaces (Lei et al., 2022).]”.

The reference Singh et al. 2011 (already present in the reference list) should replace the reference Savino et al. 2011 in the text. The citation has now been inserted in section [4. **Discussion**, last Paragraph] and should read: “[(“The evidenced anti-biofilm formation of 11S and M11S against gram-negative (particularly *K. oxytoca*) and gram-positive bacteria (*S. aureus*) is a significant result since the first one is known as an opportunistic and clinically dangerous pathogen (Singh et al., 2011)”).]”.

The reference Singh et al. 2011 should be eliminated from the text [**4. Discussion**, Paragraph before last] and the concerned text should read [(This action resulted probably from a quick adhesion of the tested proteins (11S and M11S) on the surface of Petri dishes due to their cationic nature, preventing the bacterial adhesion, i.e., increased protein adsorption on the solid surface reduced the bacterial adhesion.)].

The references McLaughlin et al. (2018), Dourado et al. (2019), Hsu et al. (2023), and Savino et al. (2011), Jeyasekaran et al. (2003), Zhang et al. 2022 are removed from the text and the reference list.

The reference Jeyasekaran et al. (2003) was incorrectly cited for the quantitative biofilm-formation assay. It should be replaced by Adukwu et al. (2012).

“[Adukwu, E. C., Allen, S. C., and Phillips, C. A. (2012). The anti-biofilm activity of lemongrass (*Cymbopogon flexuosus*) and grapefruit (*Citrus paradisi*) essential oils against five strains of *Staphylococcus aureus*. *J. Appl. Microbiol*. 113, 1217–1227. doi: 10.1111/j.1365-2672.2012.05418.x].”

The relevant text (section 2.2.8., first sentence) now reads “(The quantitative biofilm formation by both *S. aureus* and *K. oxytoca* was assayed using the protocol according to Adukwu et al. (2012).”

The reference Zhang et al. (2022) was inappropriately cited in the **Discussion** section (Paragraph 8) and should be replaced by “Li, Y., Wang, S., Xing, Z., Niu, Y., Liao, Z., Lu, Y., et al. (2022). Destructing biofilms by cationic dextran through phase transition. *Carbohydr. Polym*. 279:118778. doi: 10.1016/j.carbpol.2021.118778”

The relevant text should now reads: “Alternatively, the cationic M11S and 11S could have affected biofilm formation by disrupting the gel formed by the extracellular polymeric substances. This is supported by Li et al. (2022), which demonstrated that cationic dextrans and polyethyleneimine (PEI) can disrupt *P. aeruginosa* biofilms by inducing a gel-to-sol phase transition of the biofilm matrix.”

- Although the published Figure 2 accurately represents the underlying experimental data, we respectfully request that the published record be clarified to state that the untreated control growth curves were intentionally used as a common experimental baseline for both publications because both studies originated from the same experimental series, whereas the treatment groups and the scientific objectives differed between the two manuscripts. This clarification does not alter the experimental data, the interpretation of the results, or the conclusions of the article. Rather, it is intended to improve transparency and provide readers with a clearer understanding of the relationship between the two publications.

A correction has been made to **Results**, ***3.2.2 Inhibition of liquid bacterial*** ***growth***.

“The data in Figure 2, representing the 24-h growth curves of seven pathogenic bacteria subjected to one MIC of 11S and M11S, show general substance-based growth inhibition. In the liquid media, 1.0 MIC of both agents was nearly sufficient to completely prevent the 24-h liquid bacterial growth of all the tested microorganisms. *Listeria monocytogenes* and *K. oxytoca* were the most inhibited organisms. The untreated control and 11S growth datasets' curves were intentionally shared between the two publications (Abdel-Shafi et al., 2022 and Enan et al., 2023, retracted) as they originated from the same experimental series, as a common experimental baseline for both. Alternatively, the two treatment groups datasets (BS and M11S) were unique for each of (Abdel-Shafi et al., 2022, retracted and Enan et al., 2023), respectively. Additionally, the scientific objectives differed totally between the two manuscripts.” It should be clarified that the retraction of the first publication (Abdel-Shafi et al., 2022, retracted) has no bearing on the experimental data reused in the present article. The reuse of these shared controls was not disclosed in the original manuscript since the two articles were nearly prepared at the same time.

- The published wording describing the interpretation of the ANOVA test is incorrect. The published text inadvertently states that rejection of the null hypothesis (p < 0.05) indicates the absence of significant differences between the means.

The correct interpretation is:

• If *p* < 0.05, the null hypothesis is rejected, indicating statistically significant differences among the group means.

• If *p* ≥ 0.05, the null hypothesis is not rejected, indicating that no statistically significant differences were detected.

A correction has been made to **Materials and Methods**, ***2.2.10 (Statistical*** ***analysis)***

“SPSS program version 23 was used to statistically analyze the mean and standard deviation data. Two-way ANOVA tests were performed to compare the different microorganisms (i.e., *L. monocytogenes, K. oxytoca, L. ivanovii, S. aureus, P. mirabilis, P. aeruginosa, and S. typhimurium*), as well as the different concentrations, (i.e., 0.05, 0.1, 0.3, 0.5, 1, 2, 4, and 8 μg/ml for 11S and 0.0125, 0.025, 0.05, 0.1, 0.3, and 0.5 μg/ml for M11S), and the interactions between them for inhibition zone diameter. The null hypothesis was rejected if the ANOVA *p*-value was < 0.05, indicating statistically significant differences among the group means. When the *p*-value was ≥0.05, the null hypothesis was not rejected, indicating that no statistically significant differences were detected. The two-way ANOVA test with a *post-hoc* test using Duncan's test was applied to make multiple comparisons between the averages of different groups. Means followed by the same letter were not significantly different at the 5% probability level (Duncan's multiple range tests). The results were presented as the means of three replicates ± SD. The statistical analyses which were conducted using two-way ANOVA and Duncan's multiple-range test accurately reflect the methods applied to the original datasets.”

- There was a mismatch between the SEM method mentioned that 30 kV while in Figure 5, which also contained a few pictures taken at 25 kV. The method section (***2.2.9. Scanning electron microscopy of bacterial biofilm***, the last phrase) complies now with the figure and reads (before coating with gold–palladium and observation at 25 and 30 kV).
- There is a typo error in the **Results** (section 3.2.7.), which is now corrected and reads (thus destroying the biofilm after 24 h of incubation at 37 °C.)
- There was a typo or copy-paste error during the handling of data in Table 1. Therefore, the mean concentration effect at this concentration must be **0.00** **±** **0.00**, not **7.429** **±** **0** which should be moved to the right. The other values and the corrected values in the last row should read as follows:

**Table 1:** 

	0.0125	0.025	0.05	0.1	0.3	0.5
Concentration effect	0.00 ± 0	7.429e ± 0	8.28d ± 12.5	17.42c ± 13.7	21.28b ± 13.5	26.85a ± 13.3

In response to an enquiry comment on the interpretation of NMR and FTIR analysis we revised the relevant section and induced the following clarifications and corrections to eliminate typographic errors and ambiguity.

We have corrected the integration error at 1.51 ppm from (1H) to (3H). The right diagnostic signal for a carboxyl methyl ester COOCH_3_ appeared at 3.6–3.8 ppm, while the peak at 2.68 ppm has been removed from the text as it is irrelevant.

The units used to describe the 7.50 → 8.58 shift have been corrected from Hz to ppm throughout the manuscript The signal describing “imine” should be changed to amide (included in the peptide bond), consistent with a protein backbone environment.

The comment on the 11S NMR spectrum should be clarified to read. The 11S globulin spectrum, containing ~6 discrete NMR resonances with clean integer proton integrations, indicated the true complexity of a heterogeneous, high-molecular-weight protein.

Regarding the qualitative nature of the FTIR evidence, we should clarify that we conducted other quantitative experimental analyses to consolidate the reliability of our esterification claims. We mentioned that the data in Figure 1A confirm that esterifying lupine 11S globulins accelerated their migration toward into the cathode in Urea-PAGE. This change was further confirmed by tracing the pH–solubility curves of both the native (11S) and esterified protein (M11S), indicating that the isoelectric point of the methylated form (M11S) was relatively higher (pH 8.8) than that of the native form (11S), i.e., pH 7.3.

The original version of this article has been updated.
