# Supplementary figures and images for: Controlling bacterial biofilm formation by native and methylated lupine 11S globulins
Source: Front Microbiol. 2023 Sep 26;14:1259334. doi: 10.3389/fmicb.2023.1259334 (PMC10562546; doi:10.3389/fmicb.2023.1259334)

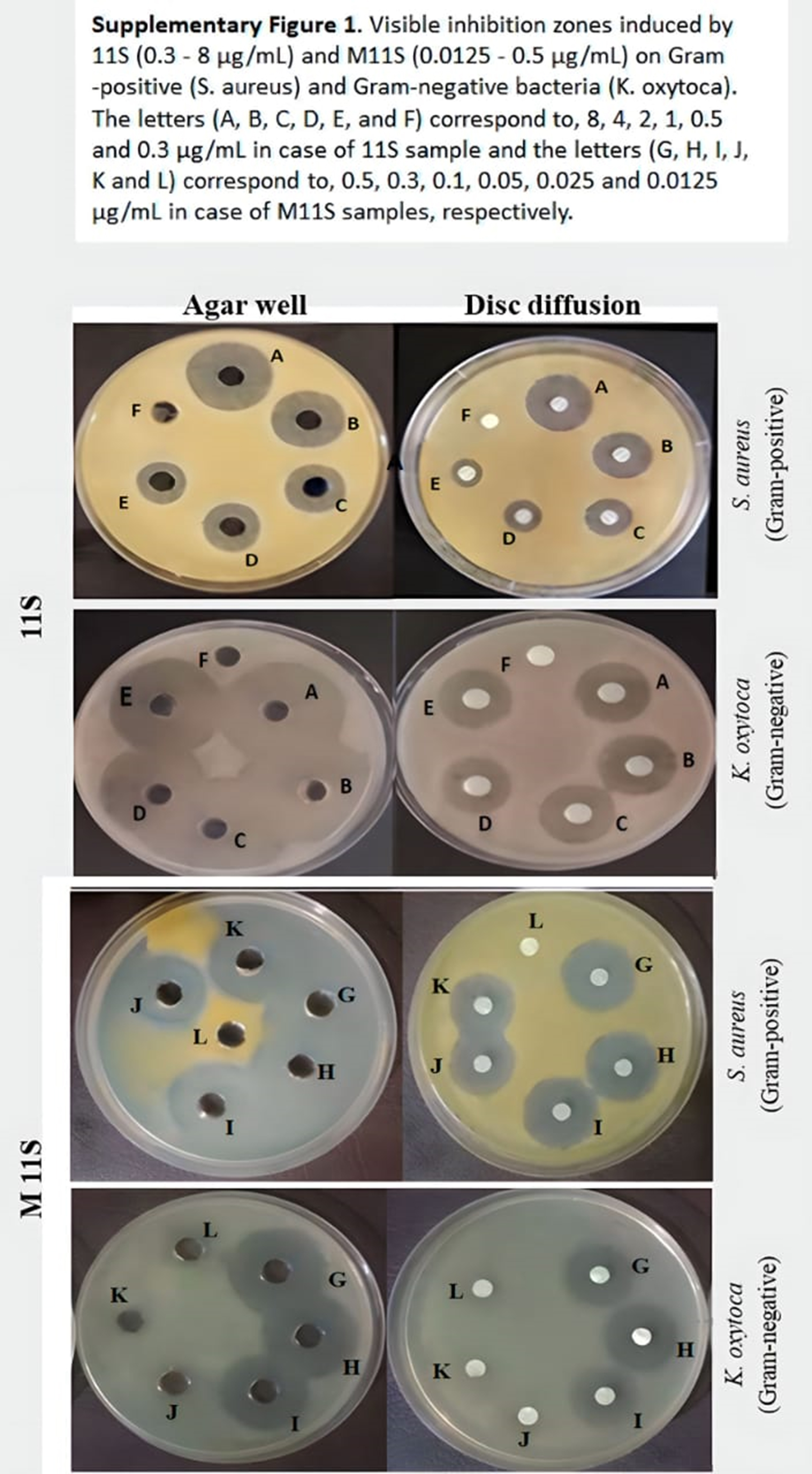

Supplement: Supplementary file 1 [file Image_1.JPEG]

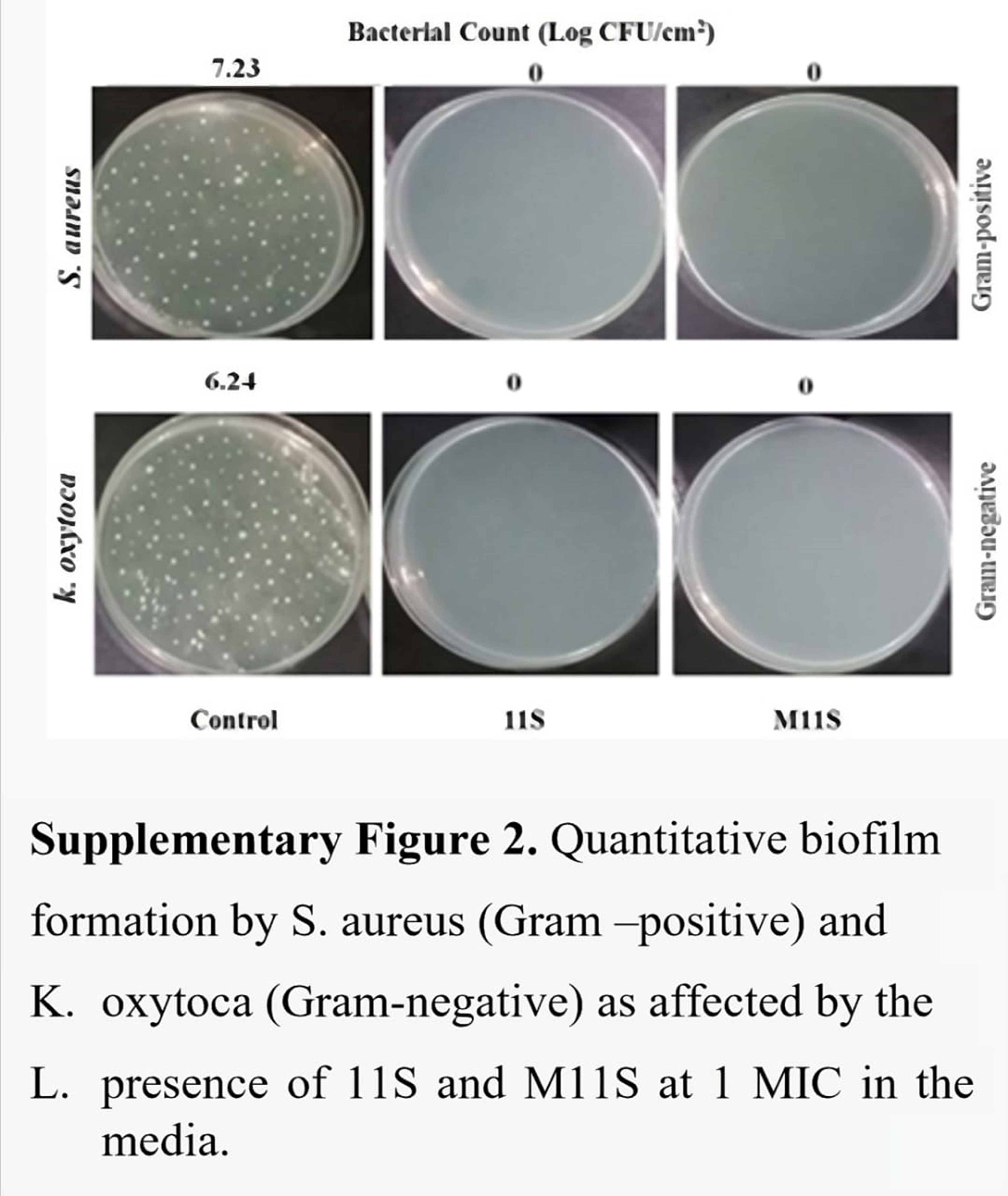

Supplement: Supplementary file 2 [file Image_2.JPEG]
